# FOXK2 Transcription Factor and Its Emerging Roles in Cancer

**DOI:** 10.3390/cancers11030393

**Published:** 2019-03-20

**Authors:** Gabriela Nestal de Moraes, Luciana da Torre Carneiro, Raquel Ciuvalschi Maia, Eric Wing-Fai Lam, Andrew David Sharrocks

**Affiliations:** 1 Laboratório de Hemato-Oncologia Celular e Molecular, Programa de Hemato-Oncologia Molecular, Instituto Nacional do Câncer (INCA), Praça da Cruz Vermelha, 23, 6° andar, Rio de Janeiro (RJ) 20230 130, Brazil; lucianadatorre@gmail.com (L.d.T.C.); rcmaia@inca.gov.br (R.C.M.); 2 Department of Surgery and Cancer, Imperial College London, Hammersmith Hospital Campus, Du Cane Road, London W12 0NN, UK; eric.lam@imperial.ac.uk; 3 Faculty of Biology, Medicine and Health, University of Manchester, Oxford Rd, Manchester M13 9PL, UK; andrew.d.sharrocks@manchester.ac.uk

**Keywords:** FOXK2 transcription factor, forkhead box, transcriptional activity, cancer

## Abstract

Forkhead box (FOX) transcription factors compose a large family of regulators of key biological processes within a cell. FOXK2 is a member of FOX family, whose biological functions remain relatively unexplored, despite its description in the early nineties. More recently, growing evidence has been pointing towards a role of FOXK2 in cancer, which is likely to be context-dependent and tumour-specific. Here, we provide an overview of important aspects concerning the mechanisms of regulation of FOXK2 expression and function, as well as its complex interactions at the chromatin level, which orchestrate how it differentially regulates the expression of gene targets in pathophysiology. Particularly, we explore the emerging functions of FOXK2 as a regulator of a broad range of cancer features, such as cell proliferation and survival, DNA damage, metabolism, migration, invasion and metastasis. Finally, we discuss the prognostic value of assessing FOXK2 expression in cancer patients and how it can be potentially targeted for future anticancer interventions.

## 1. FOXK2 Transcription Factor

**Figure 1:**
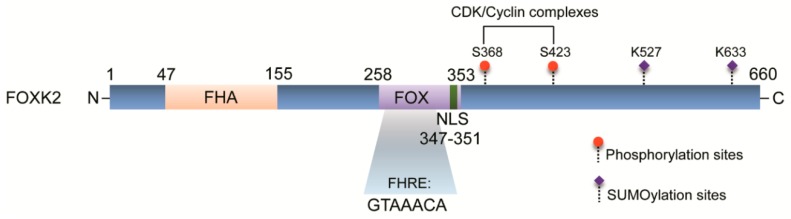
The domain structure of FOXK2 protein. FOXK2 is composed of a forkhead-associated domain (FHA) localized towards the amino terminus and a highly conserved forkhead DNA-binding domain (FOX) localized towards the carboxy-terminal end of the protein. The FOX domain displays a nuclear localization signal (NLS) and mediates FOXK2 binding to consensus sequences with a GTAAACA core motif. The post-translational modifications (PTM) for FOXK2 are shown, where serines (S) are targets for phosphorylation by cyclin-dependent kinases (CDK) and lysines (K), for SUMOylation. Amino-acids sites which are targets for PTM are depicted above the scheme. FHRE, forkhead responsive elements.

FOXK2 is a transcription factor belonging to the forkhead box (FOX) family. Members of this family share an evolutionary conserved winged helix DNA binding domain [1] and are known to regulate a wide range of biological processes including metabolism, cell cycle progression, proliferation, survival, differentiation and apoptosis [2]. FOXK2 was initially described as an interleukin-enhancer binding factor (ILF), in a search for genes encoding cellular factors which bind to purine-rich motifs in the human immunodeficiency virus (HIV) long terminal repeat [3]. In this study, FOXK2 was shown to bind to the *interleukin-2* (*IL-2*) promoter, the reason why its gene was initially designated *ILF1*. Although FOXK2 can occupy the *IL-2* promoter, it does not directly interact with components of nuclear factor of activating cells (NFAT) complex [4], a transcription factor critically required for IL-2 mRNA synthesis in activated T-lymphocytes. This further suggests that FOXK2 might help recruit cellular factors to the *IL-2* promoter in resting cells or maybe alter the chromatin structure to enable the binding of NFAT proteins as a preceding step for transcriptional activation. The *FOXK2* gene is located on human chromosome 17q25.3 and transcribes an mRNA expressed in a variety of tissues [5]. FOXK2 mRNA undergoes alternative splicing, with three isoforms having been identified to date [4,5], whose functional differences have not been explored. Although *FOXK2* gene mutations have not been reported, a partial tetrasomy of 17q25 chromosome disrupting the *FOXK2* gene has been associated with intellectual disability, malformations, syndactyly and central nervous system abnormalities in a 10-year old child [6], suggesting a possible role in development. The *FOXK2* gene has also been found to be hypomethylated in CpG islands in obese patients adipose tissues, which associates with high levels of nicotinamide N-methyltransferase (NNMT), the major methyltransferase in this tissue type [7]. Following weight loss, the patients exhibited decreased NNMT levels, along with changes in the expression of FOXK2 and some inflammation-related genes [7]. This finding indicates that *FOXK2* gene expression could be modulated by epigenetic modifications, and suggests that FOXK2 might play pathophysiological functions in adipocyte tissue metabolism. FOXK2 is a 660 amino acid protein, structurally characterized by a forkhead-associated (FHA) domain, a FOX domain and a nuclear localization signal (NLS) (Figure 1). FHA domains are phosphoprotein-interacting motifs present in prokaryotes and eukaryotes, which specifically recognize phosphorylated residues, particularly threonine and serine [8]. Therefore, FHA-containing proteins are generally involved in cell signaling cascades, such as the DNA damage checkpoint response, cell growth, signal transduction and cell cycle regulation [9]. The FHA domain is not restricted to FOXK2, being also found in the FOXK1 protein, the other member of FOXK subfamily in humans. FOXK1 and FOXK2 present a high similarity in protein sequence, sharing almost 50% total amino-acid identity [10]. In comparison to its subfamily member FOXK1, much less is known about FOXK2 biology and how it impacts the transcriptional landscape. 

## 2. Mechanistic Insights into FOXK2 Transcriptional Activity

Although FOXK2 was identified in the early nineties [3], its biological roles have remained unrevealed for almost two decades. Only in the last ten years have insights into the functions of FOXK2 been published, which indicate that FOXK2 plays many broad and distinct roles and is involved in multiple molecular signaling pathways within the cell.

### 2.1. Chromatin Binding Specificities of FOXK2

**Figure 2:**
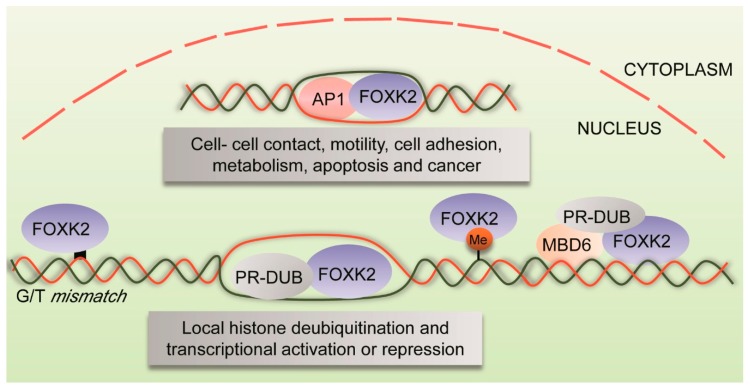
FOXK2 transcription factor and chromatin-associated events. FOXK2 recruits AP-1 to the chromatin, regulating genes involved in cell-cell contact, motility, cell adhesion, metabolism, apoptosis and cancer. Besides that, FOXK2 interacts with BAP1, a component of PR-DUB complex, leading to local histone deubiquitination and to repression and/or activation of transcription of target genes. In addition to these chromatin-associated events, FOXK2 binds to methylated DNA as well as interacts with MBD6 and PR-DUB complex, recruiting transcriptional complexes. Furthermore, FOXK2 is potentially implicated in DNA repair functions, where it binds to G/T-mismatch regions and interacts with BAP1 deubiquitinase. BAP1, BRCA1-associated protein 1; AP-1, activator protein-1; PR-DUB, polycomb repressive deubiquitinase; MBD6, methyl binding domain protein 6; Me, DNA methylation sites.

FOXK2 binds to consensus sequences with a GTAAACA core motif, which is quite similar to motifs recognized by other FOX proteins (Figure 1). Despite sharing the FOX domain with other family members, the specificity and differential binding to target genes are thought to be influenced by the flanking regions [4,11] and, importantly, by the complex interactions with other transcription factors at the chromatin level (Table 1). Indeed, a model of dynamic partial occupancy of binding regions has been determined for FOXK2 and FOXO3a, which have been shown to bind in a widespread manner to the same genomic locations, and thereby govern FOXO-dependent gene expression in U2OS cells [11]. In this scenario, rather than directly competing for chromatin occupancy, FOXK2 and other FOX proteins are thought to dynamically associate and dissociate from their shared binding motifs and only regulate targets when other appropriate co-regulatory partners are present. However, genome-wide chromatin immunoprecipitation (ChIP)-sequencing analysis has revealed that FOXK2 occupies unique binding regions throughout the genome in human cells, despite sharing some binding sites with other FOX factors [12]. Most of these regions have been shown to be associated with transcriptionally active genes which are either repressed or activated respectively, following functional assays involving FOXK2 knockdown and overexpression. These target genes are enriched for components of signaling pathways associated with cell adhesion and motility, control of transcription and metabolism, apoptosis and cancer (Figure 2). Interestingly, FOXK2 binding regions were found to be enriched for binding motifs of the activator protein-1 (AP-1) transcription factor, particularly in regions with open chromatin [12]. Consistent with this finding, FOXK2 was shown to functionally interact with AP-1 and promote its transcriptional activity. Conversely, FOXK2 depletion results in reduced binding of FOS component of AP-1 to chromatin, indicating that FOXK2 is required for AP-1 recruitment and transcription of targets genes. In this context, FOXK2 appears to be acting towards promoting transcriptional activation. Considering that the AP-1 transcription factor regulates transcription in response to a wide range of stimuli, controlling cellular processes particularly involved in cell fate decisions [13], FOXK2 might play a crucial role in modulating signal-associated gene expression.

### 2.2. FOXK2 and Histone Ubiquitination

In addition to AP-1, the breast cancer type 1 susceptibility protein (BRCA1)-associated protein 1 (BAP1) deubiquitinase was subsequently identified as a partner for FOXK2 in human cells [14]. BAP1 is an important tumour suppressor and a component of the polycomb repressive deubiquitinase (PR-DUB) complex, known to catalyze histone H2A deubiquitination [22,23] (Figure 2). The BAP1-FOXK2 interaction is abrogated with a mutant version of FOXK2 with impaired phosphopeptide binding activity, suggesting a dependency on the FHA domain of FOXK2 [14]. FOXK2 is required for BAP1 recruitment to chromatin, where they have been shown to bind to the same genomic regions and promote local histone deubiquination to regulate transcription, by both repressing and activating target genes [14]. In cell line models of human embryonic kidney and lung cancer, FOXK2-mediated recruitment of BAP1 to target genes has been confirmed by another study, which identified Thr493 as the phosphorylation site of BAP-1 and that this site is necessary for interaction with the FHA domain of FOXK2 [24]. Also, BAP1 is required to form a ternary complex with FOXK1/K2 and HCF-1 and suppresses activation of FOXK2 target genes dependent on ubiquitination of Ring1B, a ubiquitin E3 ligase [24].

### 2.3. FOXK2 and Cell Metabolism

#### 2.3.1. FOXK2 and Autophagy

**Figure 3:**
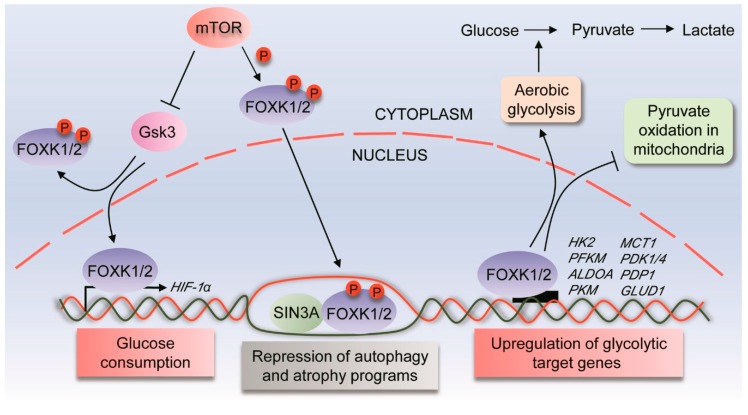
FOXK1/2 transcription factors in the control of metabolic processes. FOXK1/2 transcription factors are phosphorylated by mTOR, which translocate them to the nucleus where they interact with the Sin3A complex to suppress genes involved in atrophy and autophagy programs. Moreover, mTOR induces nuclear translocation of FOXK transcription factors through GSK regulation. In this context, FOXK1/2 induces *HIF1α* gene expression and glucose consumption, contributing to cell proliferation. Additionally, FOXK1/2 regulate aerobic glycolysis while suppressing aerobic oxidation through the upregulation of glycolytic target genes. mTOR, mammalian target of rapamycin; Gsk3, glycogen synthase kinase 3; HIF1α, hypoxia-inducing factor 1 alpha; SIN3A complex, SIN3 Transcription Regulator Family Member A complex; HK2, hexokinase-2; PFKM, phosphofructokinase muscle isoform; ALDOA, aldolase A; PKM, pyruvate kinase M1/2; MCT1, monocarboxylate transporter 1; PDK1/4, pyruvate dehydrogenase kinases 1/4; PDP1, Pyruvate dehydrogenase phosphatase 1; GLUD1, glutamate dehydrogenase 1; P, phosphorylation site.

FOXK2 has also been shown to interact with proteins from the Sin3A histone deacetylase (HDAC) co-repression complex in human cells [14], suggesting another mechanism underlying FOXK2 regulation of transcription (Figure 3). In this scenario, FOXK2 would be expected to act as a transcriptional repressor protein. Further studies in mouse myoblasts and human fibroblasts have also shown that FOXK2 and the closely related FOXK1 can associate with Sin3A, but not with the related Sin3B containing complex. This study went on to further implicate FOXK factors in the regulation of autophagy programs and thus, metabolism [15]. In this context, FOXK1/2 is recruited to and represses genes involved in atrophy and in the initiation of autophagy and vesicle nucleation, such as *Ulk1*, *Ulk2*, *Vps34*, *Ambra1* and *Atg13*, a condition that is reversed upon starvation [15]. Mechanistically, FOXK1-Sin3A repressive interactions at the chromatin level locally modify the histone acetylation profile and the structure of nucleosomes, in a process which involves an exclusive interplay with FOXO3a transcription factor, previously shown to activate autophagic genes in mouse myoblasts [25]. Although interactions of FOXK members with Sin3B were not confirmed in this study, the association of FOXK1 with Sin3a and Sin3b complexes had been previously demonstrated by others in C2C12 myoblasts and NIH 3T3 cells [26,27], suggesting that the recruitment and formation of FOXK-Sin3 complexes on chromatin might be cell-specific or depend on other proteins in the complex. FOXK1-mediated repression of autophagy is facilitated by the mammalian target of rapamycin (mTOR) (Figure 3), a key regulator of cell homeostasis, while a mutant form of FOXK1 defective for mTOR phosphorylation is relocated to the cytoplasm and displaced from target genes [15]. The role of FOXK transcription factors in metabolism under mTOR control has been subsequently explored in a study which implicated FOXK1/2 in the regulation of glucose consumption [28]. FOXK1/2 knockdown leads to downregulation of *hypoxia-inducing factor 1 alpha (HIF1α)* gene expression and suppression of cell proliferation of mouse embryonic fibroblasts [28]. FOXK1 has been shown to link mTOR signaling to HIF1α-mediated transcription, through the direct phosphorylation of FOXK1 by glycogen synthase kinase 3 (Gsk3) (Figure 3). Although most of the experiments in these studies have been performed with FOXK1 rather than FOXK2, FOXK1 and FOXK2 were shown to bind to the same regulatory regions associated with their target genes, which might be expected as they share 91% identity in their DNA binding domains [15]. Also, residues around mTOR-regulated phosphorylation sites are highly conserved between FOXK1 and FOXK2, which suggests that FOXK2 might have its subcellular localization modulated by mTOR [15,28]. Additionally, FOXK2 depletion resulted in de-repression of FOXK1 [15], which suggests that FOXK1 and FOXK2 might play redundant roles in the transcriptional regulation of some target genes.

#### 2.3.2. FOXK2 and Aerobic Glycolysis

More recently, a study has implicated FOXK2 in the regulation of aerobic glycolysis [29]. FOXK1 and FOXK2 expression levels were found to be upregulated in muscle and adipocyte tissues of starved mice, as well as in adipocytes of obese mice and skeletal muscle following exercise [29]. In response to FOXK1 and FOXK2 overexpression, glucose uptake was increased in myoblasts and myotubes, an effect that was reversed by FOXK1/2 knockdown. Furthermore, glycolysis was induced in adipocytes following overexpression of FOXK1/2, demonstrating that they are key transcriptional regulators of glycolytic enzymes. This effect is accompanied by an increase in lactate production and suppression of pyruvate oxidation in the mitochondria (Figure 3). Interestingly, transcriptome analysis of adipocytes with FOXK1/2 overexpression and knockdown revealed that differentially expressed glycolytic pathway genes were co-regulated by both FOXK1 and FOXK2, pointing to additional redundancies in their functions. The in vivo relevance of these findings was confirmed, as mice lacking *FOXK2* expression exhibited decreased glucose uptake in several tissues [29], further implicating FOXK2 as a potent regulator of aerobic glycolysis. 

Altogether, these studies clearly attribute a role for FOXK transcription factors in the control of metabolic processes and therefore, in maintaining cellular homeostasis. Further research is required to uncover not only the potential unique functions that FOXK1 and FOXK2 might play, but also their redundant roles in the transcriptional control of gene networks involved in metabolism. However, we lack information on the mechanisms of regulation governing FOXK expression and function in this context and, eventually, in pathological conditions.

### 2.4. FOXK2 and DNA Methylation

Besides the aforementioned chromatin-associated events, FOXK2 has been demonstrated to bind to methylated DNA in mouse embryonic stem cells [30,31]. Data concerning the preferential DNA modification recruiting FOXK2 is controversial, with a report pointing to 5-methylcytosine [30] and another, to its oxidized derivative 5-formylcytosine [31]. Although the effects on transcription have not been assigned, FOXK2 has been suggested to act as a methyl binding domain protein (MBD), known to be responsible for the readout of DNA methylation and for coordinating this with other modifications to regulate transcription, replication and DNA repair [32]. The mechanisms by which DNA methylation regulates transcription are still poorly understood, but a report has demonstrated that FOXK2 functionally interacts with MBD6 and PR-DUB in human cells [16], establishing a link between DNA methylation and the recruitment of transcriptional complexes (Figure 2). In this study, MBD5 and MBD6 have been described as novel interactors of PR-DUB, in an MBD domain-dependent manner. On the other hand, MBD6, but not MBD5, is recruited to sites of laser-induced DNA damage independently of its MBD domain and PR-DUB interaction [16]. Altogether, these findings implicate FOXK2 in rather complex chromatin-mediated events, including histone acetylation and ubiquitination and DNA methylation, which finely modulate FOXK2 roles as both a repressor and activator of gene transcription.

## 3. FOXK2 in DNA Repair and Cell Cycle Control

Three of the underlying molecular drivers of cancer are alterations to the gene regulatory machinery, disruptions to the cell cycle control machinery and impairment of the DNA repair process. As FOXK2 has been shown to be associated with all of these processes, it is an attractive candidate for involvement in tumorigenesis.

### 3.1. FOXK2 and DNA Repair

One of the first descriptions of FOXK2 functions was the finding that it could act as a novel G/T-mismatch specific binding protein (nGTBP) [33]. FOXK2 has been shown to bind to mismatched DNA through the FOX domain with high specificity and affinity in human cells [33] (Figure 2), suggesting that it might sense G/T mismatches and trigger mechanisms of DNA repair. Considering that repair of DNA mismatches is a crucial step in preventing mutations [34], FOXK2 might work ensuring replication fidelity and genomic stability. Interestingly, FOXK2 does not contain a catalytic domain usually found in DNA repair proteins. So, it is reasonable to hypothesize that it can recognize and interact with phosphoproteins involved in DNA repair cascades via its FHA domain. Indeed, FOXK2 has been shown to physically interact with BAP1 [14], a protein implicated in DNA repair in addition to its widely studied role in gene regulation [35]. BAP1 phosphorylation and its own catalytic activity are required for its recruitment to double-strand DNA break lesions and promotion of DNA repair by homologous recombination [35]. This suggests that FOXK2 and BAP1 might act in concert and be involved not only in chromatin remodeling and gene expression control, but also in DNA repair pathways. Altogether, these findings potentially imply a function for FOXK2 in the context of the DNA repair regulatory machinery. FOXK2 might be engaged in DNA repair functions by binding to sites of DNA lesions, both interacting with molecules in the DNA repair cascade, potentially via its FHA domain, and recruiting complexes involved in the initiation of the DNA repair signaling pathway. Interestingly, the yeast forkhead protein Fkh1, the homolog of human FOXK1, has been found to be associated with chromatin following induction of DNA double-strand breaks and to regulate donor preference in yeast in an FHA domain-dependent manner [36].

### 3.2. FOXK2 and Cell Cycle Regulation

**Figure 4:**
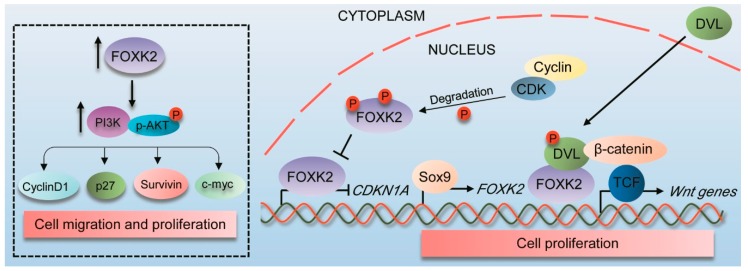
FOXK2 role as an oncogene in cancer. FOXK2 is transcriptionally regulated by the *Sox9* oncogene and promotes proliferation of colorectal cancer cell lines. In this context, FOXK2 interacts with DVL, translocating it to the nucleus, and then promoting Wnt/β-catenin signaling pathway. In agreement, FOXK2 overexpression promotes cell migration and proliferation in hepatocellular carcinoma, which is associated with high levels of Survivin, c-Myc, p27, cyclin D1 and phosphorylated AKT protein expression. A role in cell cycle progression has also been attributed to FOXK2, which is phosphorylated by CDK-cyclin complexes in a cell cycle dependent manner, in a process that induces its degradation and impairs its transcriptional activity following cell division. Box represents associations between FOXK2 and other molecules in studies involving overexpression and inhibition of FOXK2. PI3K, phosphoinositide 3-kinase; AKT, protein kinase B; Cyclin D1, regulator of cell cycle progression; p27, a cell cycle inhibitor; Survivin, a member of the inhibitor of apoptosis proteins (IAPs) family; c-myc, proto-oncogene transcription factor; DVL, Dishevelled; Sox9, the sex-determining region Y box 9; TCF, T-Cell Factor; CDK, cyclin-dependent kinase; *CDKN1A*, gene encoding p21, a cell cycle inhibitor; P, phosphorylation site.

Beyond the function in DNA repair, a role in cell cycle progression was attributed to the yeast forkhead proteins Fkh1 and Fkh2, homologs of human FOXK1 and FOXK2 [37]. Fkh2 activity has been associated with pseudohyphal growth, in which it has been shown to transcriptionally regulate genes involved in the M-phase transition [37]. Additionally, it has been demonstrated that Fkh2 is regulated in a cell-cycle dependent basis, being phosphorylated by Cdc28p, the major cyclin-dependent kinase in yeast [38]. Fkh2 phosphorylation appears to stabilize interactions of Fkh2 complexes with the co-activator protein Ndd1 at the chromatin level, directly controlling cell-cycle associated gene expression and activating transcription [38]. As Ndd1 is a target for Cdc5 (Plk1 in mammalian cells) [39], Fkh2 acts as a focal point for integrating cell cycle-dependent signals into a transcriptional response. Accordingly, FOXK2 has been identified as a target for CDK-cyclin complexes in human cells, in a study which reported that FOXK2 levels fluctuate in a cell cycle dependent manner, peaking during M phase [17]. Although located in the nucleus of asynchronous U2OS osteosarcoma cells, FOXK2 is excluded from DNA just following cell division, which is linked to decreased binding to the promoter of *MCM3* target gene, a key component of pre-replication complex [17]. In this study, serines 368 and 423 were identified as the major residues for FOXK2 phosphorylation by CDK/cyclin complexes (Figure 1), particularly CDK1 and cyclin B. CDK-mediated phosphorylation affects FOXK2 stability, induces its degradation and impairs its transcriptional activity (Figure 4) [17], providing the first insights into the role of post-translational modifications in the regulation of FOXK2 expression and function. Interestingly, mice lacking MNF—the mouse homologue of FOXK1—show defects in muscle development and regeneration following injury [40]. This further suggests that MNF/FOXK1 regulates genes involved in differentiation and/or proliferation of myogenic stem cells, thus being crucial for the determination of cell fate decisions. Of note, FOXK2 mRNA levels are upregulated upon skeletal muscle vibration stress in humans [41], indicating that there is much to be discovered on the relationship between FOXK transcription factors and muscular physiology.

## 4. Tumour-Specific Roles of FOXK2 as Either an Oncogene or Tumour Suppressor

**Figure 5:**
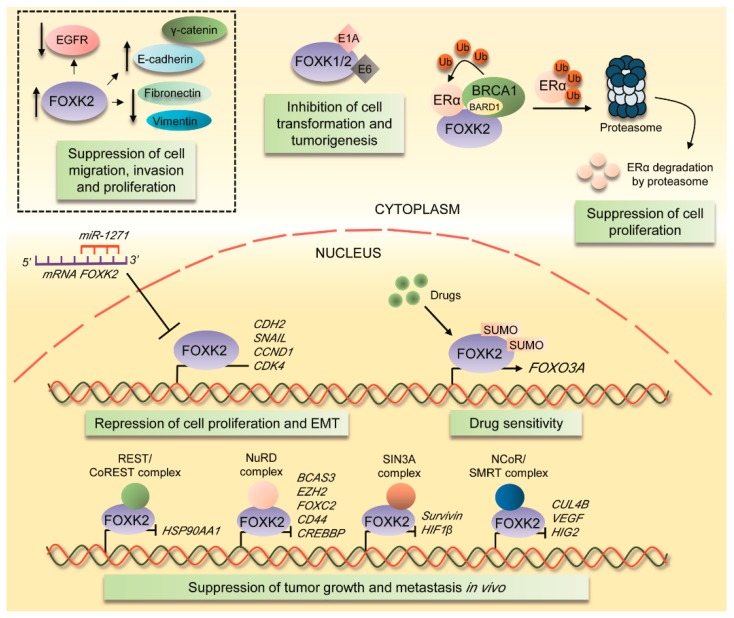
FOXK2 role as a tumor suppressor in cancer. FOXK transcription factors interact with E1A adenovirus proteins and E6 proteins from human papillomaviruses 21/14 suppressing malignant transformation. In breast cancer, FOXK2 acts as a scaffold protein between ERα and BARD1 leading to ubiquitin-mediated degradation of ERα and suppression of cell proliferation. Besides that, FOXK2 interacts with REST/CoREST, NuRD, SIN3A and NCoR/SMRT corepressor complexes to specifically repress different set of genes, inhibiting breast cancer tumorigenesis. Additionally, FOXK2 mediates the cytotoxic effects of chemotherapeutic agents in breast cancer cells through the induction of FOXO3a expression, a process stimulated by SUMOylation of specific lysine residues in FOXK2 sequence. Consistently with the descriptions of FOXK2 as a tumor suppressor, it represses *CDH2, SNAIL, CCND1* and *CDK4* genes to inhibit cell migration, invasion and proliferation in lung cancer, in which it has been described as a direct target for the miR-1271. Besides that, FOXK2 also suppresses tumorigenesis in clear-cell renal cell carcinoma, which is associated with decreased protein and mRNA levels of *EGFR* gene. Accordingly, FOXK2 overexpression is accompanied by an increase in epithelial markers and decrease in mesenchymal markers in some tumor models. Box represents associations between FOXK2 and other molecules in studies involving overexpression and inhibition of FOXK2. EGFR, epidermal growth factor receptor; γ-catenin and E-cadherin, epithelial markers; fibronectin and vimentin, mesenchymal markers; ERα, estrogen receptor-α; BARD1, BRCA1-associated RING domain protein 1; BRCA1, breast cancer type 1 susceptibility protein; *CDH2*, gene encoding N-cadherin; SNAIL (or SNAI1), snail family transcriptional repressor 1; *CCND1*, gene encoding Cyclin D1; *CDK4*, gene encoding Cyclin Dependent Kinase 4; REST/CoREST complex, RE1 Silencing Transcription Factor (REST)/REST corepressor 1(CoREST) complex; NuRD complex, nucleosome remodeling and deacetylase complex; SIN3A complex, SIN3 Transcription Regulator Family Member A complex; NCoR/SMRT complex, Nuclear receptor corepressor 1 (NCoR)/ Nuclear Receptor Corepressor 2 (SMRT) complex; HSP90AA1, heat shock protein 90 alpha family class A member 1; BCAS3, microtubule associated cell migration factor; EZH2, enhancer of zeste 2 polycomb repressive complex 2 subunit; FOXC2, forkhead box C2; CD44, cluster of differentiation 44; CREBBP, CREB binding protein; Survivin, a member of the inhibitor of apoptosis proteins (IAPs) family; HIF1β, hypoxia inducible factor 1 subunit beta; CUL4B, cullin 4B; VEGF, vascular endothelial growth factor; HIG2 (or HILPDA), hypoxia inducible lipid droplet associated; FOXO3a, forkhead box O3; EMT, epithelial-mesenchymal transition; Ub, ubiquitination; SUMO, SUMOylation site.

The first report on the role of FOXK2 in cellular transformation came from the finding that interaction of the C-terminal region of the viral E1A protein with FOXK2 is required for adenoviral-mediated suppression of tumour formation in both in vitro and in vivo assays [18]. This study demonstrated that E1A interacts with FOXK1 and FOXK2, at least in part, through their FHA domain, in a process reliant on phosphorylated E1A levels. Not only the tumour-suppressive role of E1A, but also of E6 proteins from human papillomaviruses 21/14, is likely to depend on concomitant targeting of FOXK1 and FOXK2 via a conserved Thr-Ser-containing motif [18]. Taking into account that HPV14 and HPV21, the HPV subtypes which encode the E6 protein, are linked to benign cutaneous lesions, interaction of E6 proteins with FOXK1/K2 might favor viral infection and replication in epithelial cells over the oncogenic activities of other HPVs, therefore suppressing malignant transformation (Figure 5).

The expression of FOXK2 has not been extensively addressed in normal and transformed human tissues. A report has shown that FOXK2 knockdown in non-neoplastic immortalized cell lines results in cell death and decreased proliferation and survival [42]. In the context of cancer research, evidence has emerged only in the last five years, with reports pointing to FOXK2 as a transcription factor with either oncogenic and tumour suppressing functions, as summarized in Table 2 and Table 3.

### 4.1. FOXK2 Role as a Tumour Suppressor

One of the first studies on the role of FOXK2 in cancer showed that it interacts with estrogen receptor alpha (ERα), a master regulator of breast cancer development [19]. FOXK2 interaction with ERα leads to decreased stability and ubiquitin-mediated degradation of ERα, dependently on the BRCA1-associated RING domain protein 1 (BARD1) catalytic subunit of BRCA ubiquitin ligase protein (Figure 5) [19]. FOXK2-ERα interaction is accompanied by reduced transcriptional regulation of ERα target genes and suppression of ERα-driven cell proliferation [19]. Corroborating these data, another study has shown that FOXK2 can mediate the cytotoxic effects of chemotherapeutic agents in breast cancer cells [43]. Drug sensitization is dependent on FOXK2 binding to the promoter of the gene encoding FOXO3a transcription factor, which is associated with induction of FOXO3a expression (Figure 5). Conversely, the FOXK2-FOXO3a axis is deregulated in drug-resistance cell models, in which FOXK2 fails to be recruited to *FOXO3a* promoter, despite constitutively high expression levels [43]. In this context, FOXK2-mediated cytotoxic effects in breast cancer cells have been demonstrated to be stimulated by SUMOylation (Figure 5), in a study which identified two consensus SUMOylation motifs within the FOXK2 sequence [51]. FOXK2 transcriptional activity and drug sensitivity are impaired following transfections with a form of FOXK2 mutated for lysines 527 and 633, the major sites for SUMO conjugation (Figure 1). The identification of sites of modification that play a role in controlling FOXK2 activity provides further insight into the role of post-translational modifications in FOXK2 regulation. To further explore FOXK2 functions in breast tumorigenesis, Shan and colleagues [20] have demonstrated that FOXK2 knockdown remarkably increases breast cancer cell proliferation, induces epithelial-mesenchymal transition (EMT) and enhances the invasive and migratory potential of breast cancer cells. Notably, FOXK2-depleted triple negative MDA-MB-231 cells injected in immunodeficient mice formed tumours exhibiting distant metastasis and increased growth rates, further confirming the data in the in vivo scenario [20]. FOXK2 interacts with multiple corepressor complexes, including nuclear receptor co-repressor/silencing mediator for retinoid or thyroid-hormone receptors (NCoR)/SMRT, nucleosome remodeling deacetylase (NuRD), Sin3A and RE1-silencing transcription factor (REST)/CoREST, through its FHA domain and transcriptionally represses the expression of target genes, such as *Survivin*, *BCAS3*, *CUL4B*, *EZH2*, *FOXC2, HIF1b*, *CD44*, *VEGF*, *CREBBP*, *HIG2*, and *HSP90AA1* in a complex-specific manner (Figure 5) [20]. These data implicate FOXK2 in the suppression of hypoxia and breast carcinogenesis. Lastly, this study showed that the *FOXK2* gene is transactivated by ERα, establishing a novel regulatory loop between FOXK2 and ERα and further pointing to a complex interrelationship between these proteins. It is important to highlight that FOXK2 has also been shown to play roles in triple negative models of breast cancer [20,43], suggesting that it might act independently of ER expression, through a still elusive mechanism. Altogether, this evidence strongly supports a tumour suppressive role for FOXK2 in breast cancer (Figure 5). Of note, FOXK1 has also been implicated in suppressing tumorigenesis in models of breast cancer [52], indicating that tumour suppression might be a conserved function within FOXK family members.

In agreement with the findings in breast cancer, FOXK2 has been implicated in suppressing tumorigenesis in clear-cell renal cell carcinoma [45]. FOXK2 overexpression significantly induces apoptotic features and inhibits cell growth in both in vitro and in vivo models of clear-cell renal cell carcinoma [45]. Also, the capacity of migration and invasion are impaired in FOXK2-overexpressing cells, which present decreased protein and mRNA levels of *EGFR* gene (Figure 5) [45]. This is consistent with data obtained in gastric cancer cells, where FOXK2 overexpression has been demonstrated to induce early apoptosis and inhibit cell growth, migration and invasion, which is accompanied by an E-cadherin increase and a N-cadherin decrease [46]. Similar conclusions have been derived from FOXK2 knockdown studies, where loss of FOXK2 promotes growth, invasion and migration in glioma cells, followed by modulation of expression of E-cadherin, N-cadherin and vimentin EMT markers [47]. Consistent with these findings, Chen and colleagues [44] have reported that FOXK2 overexpression in lung cancer-derived cell lines leads to upregulation of E-cadherin and α-catenin along with downregulation of N-cadherin and vimentin (Figure 5), suggesting that FOXK2 might modulate the expression of epithelial and mesenchymal markers closely linked to the EMT process. FOXK2 depletion promotes cell migration, invasion and proliferation, via transcriptional upregulation of *CDH2*, *SNAIL*, *CCND1* and *CDK4*, identified as target genes (Figure 5) [44]. Interestingly, the *FOXK2* gene has been found to be methylated in circulating leukocytes of smokers in an epigenome-wide association study with nicotine equivalents [53]. This places epigenetic modifications as additional mechanisms for silencing *FOXK2* gene expression, which might have implications for the pathogenesis of lung cancer. 

### 4.2. FOXK2 Role as an Oncogene

Despite the roles described as a tumour suppressor, a few reports have attributed an oncogenic role for FOXK2. FOXK2 has been shown to interact with nuclear Dishevelled (DVL) in colon cancer activating the Wnt/β-catenin signaling pathway [21]. FOXK2 overexpression translocates DVL to the nucleus, dependently on Wnt3a-mediated DVL phosphorylation, but not on the presence of its FOX DNA binding domain (Figure 4). FOXK2-deficient colon cancer cell lines are growth inhibited in both in vitro and in vivo settings. Conversely, FOXK2 conditional expression induces intestinal hyperproliferation [21]. Corroborating these data, Qian and colleagues [48] have reported that FOXK2 promotes proliferation of colorectal cancer cell lines and has its promoter transcriptionally regulated by the sex-determining region Y box 9 (SOX9) oncogenic protein (Figure 4) [48]. 

These findings suggest that FOXK2 might contribute to the formation of cancers originated from the colon and rectum. This is in agreement with a description on the oncogenic role for FOXK1 in colorectal cancer [54]. The gene targets involved in mediating FOXK2 oncogenic effects in colorectal cancer remain to be determined. In addition to colorectal cancer, FOXK2 has also been shown to act as an oncogene in hepatocellular carcinoma (HCC), in which it has been demonstrated to promote cell migration and proliferation with the involvement of the PI3K/AKT signaling pathway [49]. In this context, FOXK2 overexpression led to high levels of phosphorylated AKT, Survivin, c-Myc, p27 and cyclin D1 protein expression (Figure 4), an effect that was reversed following FOXK2 knockdown [49]. This study and another [44] also identified FOXK2 as a direct target for the microRNA-1271-5p [49], suggesting that FOXK2 is regulated upstream by both post transcriptional and post-translational modifications.

## 5. Prognostic Value of Assessing FOXK2 Expression in Cancer Patients

Since evidence on the role of FOXK2 in human cancer has only recently emerged, there is not much data available reporting FOXK2 expression in samples from cancer patients (Table 3). In breast cancer patient samples, FOXK2 expression has been analyzed by immunohistochemistry and around 50% positivity has been found [19,43]. In two studies, chi square tests have revealed an association between FOXK2 and ERα expression [19,43], which has not been confirmed by a third one [20]. Also, nuclear FOXK2 has been found to be associated with tumour stage and FOXO3a expression, particularly in samples from patients receiving chemotherapy [20,43]. Interestingly, FOXK2 expression is correlated with reduced disease-free survival in breast cancer patients, consistently with the in vitro data supporting the hypothesis that constitutively high expression levels of nuclear FOXK2 are closely associated with poor clinical outcome and drug resistance [43]. Nevertheless, Shan and colleagues [20] found FOXK2 expression downregulated in breast carcinomas comparatively to their adjacent tissues and correlated this with better survival curves. Some contrasting data observed in these studies might be explained by different patient cohorts and FOXK2 antibodies used, which possibly have distinct specificities and might detect diverse forms of FOXK2 protein, including still undiscovered modified forms. However, the experimental settings and the conclusions drawn in each study are supported by in vitro data and further confirm the role of FOXK2 as a tumour suppressor in breast cancer. Of note, FOXK2 and FOXO3a are evolutionarily closely linked [55] and therefore might share functional similarities, regulating the same genes and co-operating with similar transcription factors [11]. 

FOXK2 mRNA expression has been found to be downregulated in high-grade, compared to low-grade glioma [47]. Also, FOXK2 protein expression is negatively associated with tumour grade and staining of KI-67 proliferation marker and positively correlated with favorable prognosis in both uni- and multivariate analysis [47]. A positive correlation of FOXK2 protein expression with good prognosis has also been observed in gastric cancer, in which FOXK2 levels have been seemingly associated with tumour differentiation [46]. A similar phenomenon occurs in samples of non-small lung cancer patients, where FOXK2 mRNA expression is downregulated, compared with non-neoplastic tissues [44]. Consistent with these findings, high FOXK2 expression has been significantly associated with better patient outcome in non-small lung cancer [44]. This pattern of differential expression between neoplastic and non-neoplastic tissues has been confirmed in clear-cell renal cell carcinoma patients, in which FOXK2 mRNA and protein levels are downregulated compared to adjacent non-tumour renal tissues. Low FOXK2 expression is associated with worse disease-free survival and is revealed as an independent prognostic marker [45]. Contrasting this finding, *FOXK2* gene expression has been correlated with poor prognosis and associated with gender and tumour grade in a cohort of clear-cell renal cell carcinoma from The Cancer Genome Atlas (TCGA) [50]. It is therefore currently unclear whether FOXK2 functions as a tumour suppressor or oncogene in this cancer type. 

Contrasting with the data described above, FOXK2 protein expression has been found elevated in 45.5% colon carcinoma patient samples, compared to normal colon, and this is associated with nuclear DVL expression [21]. The same pattern of differential expression of FOXK2 has been found in a cohort of colorectal cancer, with a 49% high staining positivity [48]. FOXK2 expression has been linked to poor prognosis and associated with SOX9 expression, with patients co-expressing both FOXK2 and Sox9 presenting the worst survival curves [48]. Additionally, FOXK2 protein and mRNA levels have been found upregulated in hepatocellular carcinoma, compared to non-tumorous liver counterpart [49]. Significant associations have been observed between FOXK2 protein expression and clinical-pathological characteristics, such as tumour size, TNM stage and vascular invasion. Also, a negative correlation has been established between FOXK2 mRNA and the tumour suppressor miR1271 in HCC patient samples [49]. Most importantly, HCC patients presenting high FOXK2 protein expression exhibit worse survival in both uni- and multivariate analysis, pointing to FOXK2 as an independent predictor of poor prognosis in this type of cancer [49]. Altogether, these studies clearly link the FOXK2 transcription factor to a poor outcome in HCC and colorectal cancer patients. 

Overall, there is a complicated picture emerging where FOXK2 expression may have prognostic or diagnostic value but this is likely highly tumour specific and even then, may only apply to sub-types of the particular tumour and/or to particular therapeutic regimes.

## 6. Concluding Remarks and Perspectives

Further validation of the role of FOXK2 in different cancers will be necessary to establish whether FOXK2 staining or gene expression analysis might be of clinical utility as a prognostic or predictive biomarker. Likewise, the observation concerning FOXK2 differential roles in specific types of cancer is an intriguing finding, and further work on why this might be the case is needed. The recent contribution on the role of FOXK2 in the upregulation of aerobic glycolysis and glucose uptake not only in adipocytes and myocytes, but also in neuroblastoma and hypopharynx cancer cells [29], gives us insights into its function as an oncogene, particularly considering the importance of this metabolic pathway for tumour growth and metabolism. Also, the functional link between FOXK2 and autophagy [15] might potentially explain some of these findings, in face of the emerging and paradoxical roles of autophagy as either a type of cell death or a survival route in cancer, in a cell-type specific manner [56]. However, we generally lack data on the biological effects resulting from the complex interactions that FOXK2 establishes at the chromatin level. It would be noteworthy to assess, for example, classical autophagic features, such as vacuolization, LC3 lipidation and p62 staining, following disruption of FOXK2 protein interactions and binding to promoters of genes involved in autophagy. This would also apply to interactions of FOXK2 with AP-1 and BAP1, whose resultant cellular effects have not been further explored, despite unquestionable involvement in the regulation of critical gene networks [12,14]. Clearly, the confusing picture concerning the role of FOXK2 as either an oncogene or a tumour suppressor might be closely dependent on the molecular role that FOXK2 plays in each cell type and the signaling pathways with which it connects. Therefore, distinct functions may result in divergent effects in cancer, which might tip the balance according to which molecular role is dominant in a certain context. Also, FOXK2 can act as both a repressor and an activator of gene transcription depending mainly on the interaction partners and the regulated genes. Thus, a better understanding of FOXK2 upstream regulatory mechanisms as well as the identification of FOXK2 binding partners at the chromatin level and target genes regulated in each cell type might shed some light upon this issue. Also important is the assessment of similar signaling pathways across studies with different cancer types and their relationship with FOXK2 activity would be useful for direct comparison. It is reasonable to highlight that FOXK2 mRNA undergoes alternative splicing, generating three isoforms with unknown expression patterns in tissues from different origins. Therefore, it is reasonable to speculate that FOXK2 isoforms might possibly have distinct functions in different tissues, which could explain, at least in part, the dichotomous cell-dependent functions in tumorigenesis. Finally, we should not exclude the existence of as yet undiscovered potential amplifications or chromosomal translocations involving the *FOXK2* gene. Similarly, it is possible that the *FOXK2* gene is mutated in cancer, encoding either gain or loss-of-function mutant protein forms with further implications for the biology and opposing outcomes observed in different cancers.

Although there is much to be deciphered on FOXK2 biology, emerging evidence clearly links the deregulation of FOXK2 expression and function to tumorigenesis, in a cell-type specific manner. Moreover, FOXK2 has been increasingly implicated in the regulation of gene networks associated with the hallmarks of cancer, particularly those involved in cell fate decisions. Future research should attempt to uncover the missing links associated with FOXK2 roles in cancer. A better elucidation on the mechanisms of regulation of FOXK2 and how it regulates gene transcription to suppress or sustain carcinogenesis could potentially make FOXK2 an attractive target for future antineoplastic intervention.

## Figures and Tables

**Table 1:** FOXK2 interaction partners.

Interaction Partners	Technique	Interaction Effect	Models	References
FOS and JUN	IP	FOXK2 recruits components of AP-1 complex to chromatin and participates in the transcription of its target genes	U2OS and 293T cell lines	[12]
SIN3A and BAP1-containing PR-DUB complexes	RIME, co-IP and PLA assay	FOXK2 recruits BAP-1 to chromatin, promoting local histone deubiquitination and leading to activation and repression of target genes	U2OS and HEK293T cell lines	[14]
SIN3A	TAP-MS and co-IP	FOXK2 interacts with SIN3A and represses genes involved in the initiation of starvation-induced atrophy and autophagy programs	C2C12 myoblasts and IMR90 normal fibroblasts	[15]
SIN3/HDAC, NCoR, PR-DUB and NSL complexes	GFP pull down prior to MS	FOXK2/PR-DUB share target genes with MBD6, suggesting an association between FOXK2 and sites of DNA damage	293T and HeLa cell lines	[16]
CDK-Cyclin complexes	GST pull-down assay	FOXK2 is regulated by cell cycle regulatory machinery and degraded upon CDK-mediated phosphorylation	HEK293 and U2OS cell lines	[17]
E1A and E6 viral proteins	TAP-MS and co-IP	FOXK2 suppresses cell proliferation, oncogenic transformation and tumorigenesis in vivo	KB, A549, 293 and HeLa cell lines; tumours in athymic mice	[18]
ERα and BARD1	Co-IP, mammalian two hybrid system and GST pulldown assay	FOXK2 acts as a scaffold protein for ERα and BARD1, leading to a negative regulation of ERα and target genes and suppressing ERα-positive breast cancer proliferation	HEK 293T, MCF-7, Bcap-37, MDA-MB-231 and T47D cell lines	[19]
NCoR/SMRT, SIN3A, NuRD and REST/CoREST complexes	TAP-MS, co-IP and GST pull down	FOXK2 suppresses proliferation and invasion in models of breast cancer in vitro and in vivo and regulates several signaling pathways, including hypoxia	MCF-7 and HEK293T cell lines; xenograft tumours in SCID mice; breast carcinoma patients samples	[20]
DVL	TAP-MS and co-IP	FOXK2 interacts with DVL and promotes its nuclear translocation, which then activates Wnt signaling contributing to colorectal tumorigenesis	HEK293T, HeLa, HT29, DLD-1 cell lines; xenograft tumours; transgenic mice; colorectal patients samples	[21]

Rapid immunoprecipitation mass spectrometry of endogenous proteins (RIME); Immunoprecipitation (IP); Mass spectrometry (MS); Tandem affinity purification-Mass spectrometry (TAP-MS); Glutathione-S-Transferase (GST); Proximity ligation assay (PLA); Dishevelled (DVL); Cyclin-dependent kinase (CDK), Polycomb repressive deubiquitinase (PR-DUB); Estrogen receptor alpha (ERα); BRCA1-associated RING domain protein 1 (BARD1); BRCA1-associated protein 1 (BAP1); activator protein-1 (AP1); Methyl binding domain protein 6 (MBD6); Histone deacetylase (HDAC); nuclear receptor co-repressor (NCoR); non-specific lethal complex (NSL); RE1-silencing transcription factor (REST); nucleosome remodeling deacetilase (NURD).

**Table 2:** Genes regulated by FOXK2 in cancer models.

Human Cancer	Genes Regulated	Proteins Encoded	Technique	Effect on Transcription	Function	Oncogene or Tumour Suppressor	Models	References
Osteosarcoma	*CDKN1B*, *MCM3*, *CDC14A*, *KDM3A*, *KLF9*, *PDE7A* and *CAPN2*	p27, MCM3, CDC14A, KDM3A, KLF9, PDE7A and CAPN2	ChIP	Activation and repression	Regulation of gene networks involved in cell adhesion and motility, control of transcription and metabolism, apoptosis and cancer	N/A	Cell lines	[12]
Osteosarcoma	*CDKN1A*	p21	Luciferase assay	Repression	Implicated in cell cycle regulatory machinery, CDK phosphorylation of FOXK2 impacts cell viability	Oncogene	Cell lines	[17]
Breast cancer	*FOXO3A*	FOXO3a	ChIP, knockdown and overexpression	Activation	Mediates cytotoxic effects of paclitaxel and epirubicin	Tumour suppressor	Cell lines; invasive carcinoma patients samples	[43]
Breast cancer	*SURVIVIN*, *BCAS3*, *CUL4B*, *EZH2*, *FOXC2*, *HIFβ*, *CD44*, *VEGF*, *CREBBP*, *HIG2* and *HSP90AA1*	Survivin, BCAS3, CUL4B, EZH2, FOXC2, HIFβ, CD44, VEGF, CREBBP, HIG2 and HSP90AA1	ChIP-seq, qChIP and knockdown	Repression	Inhibits cell proliferation, migration and invasion in vitro and suppresses tumour growth and metastasis in vivo, mainly, by repressing hypoxia pathway	Tumour suppressor	Cell lines; xenograft tumours in SCID mice; breast carcinoma patients samples	[20]
Non-small cell lung cancer	*CDH2, SNAIL, CCND1* and *CDK4*	N-cadherin, SNAIL, Cyclin D1 and CDK4	ChIP-seq, qChIP, luciferase assays, knockdown and overexpression	Repression	Inhibits cell proliferation in part through induction of G1 arrest and suppresses EMT	Tumour suppressor	Cell lines; lung adenocarcinoma patients samples	[44]

FOXO3a, forkhead box O3; Survivin, a member of the inhibitor of apoptosis proteins (IAPs) family; EZH2, enhancer of zeste 2 polycomb repressive complex 2 subunit; BCAS3, microtubule associated cell migration factor; CUL4B, cullin 4B; FOXC2, forkhead box C2; HIF1β, hypoxia inducible factor 1 subunit beta; CD44, cluster of differentiation 44; VEGF, vascular endothelial growth factor; CREBBP, CREB binding protein; HIG2 (or HILPDA), hypoxia inducible lipid droplet associated; HSP90AA1, heat shock protein 90 alpha family class A member 1; SNAIL (or SNAI1), snail family transcriptional repressor 1; CDK4, Cyclin Dependent Kinase 4; CDKN1A—cyclin dependent kinase inhibitor 1A; MCM3, minichromosome maintenance complex component 3; CDC14A, cell division cycle 14A; KDM3A, lysine demethylase 3A; KLF9, Kruppel like factor 9; PDE7A, phosphodiesterase 7A; CAPN2, calpain 2; AXIN2, axis inhibition protein 2; LGR5, leucine rich repeat containing G protein-coupled receptor 5; ChIP, chromatin immunoprecipitation; ChIP-seq, chromatin immunoprecipitation sequencing; qChIP, quantitative chromatin immunoprecipitation; EMT, epithelial-mesenchymal transition.

**Table 3:** FOXK2 expression in samples from cancer patients.

Human Cancer	Number of Patients	Technique	% FOXK2 Overexpression	Subcellular Localization	Association with Clinical-Biological Parameters	Prognosis	Oncogene or Tumour Suppressor	Reference
Breast cancer	53 samples	IHC	47.2%	N/A	Negative association with ERα expression	N/A	Tumour suppressor	[19]
Breast cancer	86 samples	IHC	48.8%	Nuclear	Association with FOXO3a expression in samples from patients receiving chemotherapy; Association with ERα and tumour stage	Poor	Tumour suppressor	[43]
Breast cancer	140/25 samples	IHC/RT-PCR	N/A	N/A	FOXK2 mRNA and protein levels are downregulated in breast carcinomas compared to adjacent tissues; Negative correlation with EZH2 and HIF1β levels, histological grade and lymph node positivity	Favorable	Tumour suppressor	[20]
Clear-cell renal cell carcinoma	42 samples	IHC/qRT-PCR/Western blotting	50%	Nuclear	FOXK2 mRNA and protein levels are downregulated compared to adjacent non-tumour renal tissues	Favorable	Tumour suppressor	[45]
Gastric Cancer	150 samples	IHC	48.6%	N/A	FOXK2 mRNA levels downregulated in gastric cancer, compared to non-tumour tissues; FOXK2 protein expression associated with tumour differentiation	Favorable	Tumour suppressor	[46]
Glioma	151/46 samples	IHC/PCR	39.7%	N/A	FOXK2 expression downregulated in high-grade compared to low-grade gliomas and negatively associated with KI67 staining and tumour grade	Favorable	Tumour suppressor	[47]
Non-small cell lung cancer	50 samples	qRT-PCR	N/A	N/A	FOXK2 mRNA levels are downregulated in lung cancer, compared to non-tumour tissues	Favorable	Tumour suppressor	[44]
Colon cancer	200 samples	IHC	45.5%	Nuclear	FOXK2 is overexpressed compared to normal colon and associated with DVL nuclear expression	N/A	Oncogene	[21]
Colorectal Cancer	145 samples	IHC	48.9%	N/A	FOXK2 is overexpressed compared to normal tissues	Poor	Oncogene	[48]
Hepatocellular carcinoma	505/32/12 samples	IHC/qRT-PCR/Western blotting	54.1%	Nuclear	High FOXK2 expression is positively associated with tumour size, TNM stage and vascular invasion; FOXK2 mRNA levels are negatively associated with miR1271	Poor	Oncogene	[49]
Clear-cell renal cell carcinoma	525 samples	*TCGA	49.7%	N/A	*FOXK2* gene expression as an independent prognostic factor and associated with tumour grade and gender	Poor	Oncogene	[50]

DVL: Dishevelled; ERα: estrogen receptor alpha; EZH2, enhancer of zeste 2 polycomb repressive complex 2 subunit; FOXO3a, forkhead boxO3; HIF1β, hypoxia inducible factor 1 subunit beta; IHC: immunohistochemistry; qRT-PCR: Quantitative reverse transcription polymerase chain reaction; TCGA: The Cancer Genome Atlas; N/A: Not analysed.

## Abbreviations

AP-1	activator protein-1
BAP-1	BRCA1-associated protein 1
BARD-1	BRCA1-associated RING domain protein 1
BRCA-1	Breast cancer type 1 susceptibility protein
ChIP	chromatin immunoprecipitation
EMT	epithelial-mesenchymal transition
ERα	estrogen receptor alpha
EZH2	enhancer of zeste 2 polycomb repressive complex 2 subunit
FOX	forkhead BOX
FOXK1	forkhead box K1
FOXK2	forkhead box K2
FOXO3a	forkhead box O3
FHA	forkhead associated domain
Gsk3	glycogen synthase kinase 3
HCC	hepatocellular carcinoma
HIF1α	hypoxia-inducing factor 1 alpha
HIV	human immunodeficiency virus
HDAC	histone deacetylase
HPV	human papillomavirus
IL-2	interleukin-2
ILF	interleukin-enhancer binding factor
MBD	methyl binding domain protein
mTOR	mammalian target of rapamycin
NCoR/SMRT	nuclear receptor co-repressor/silencing mediator for retinoid or thyroid-hormone receptors
NFAT	nuclear factor of activating cells
NLS	nuclear localization signal
NNMT	nicotinamide N-methyltransferase
NURD	nucleosome remodeling deacetylase
PR-DUB	Polycomb repressive deubiquitinase
REST	RE1-silencing transcription factor
SOX9	Sex-determining region Y box 9
TCGA	The Cancer Genome Atlas
